# The composition of a bioprocessed shiitake (*Lentinus edodes*) mushroom mycelia and rice bran formulation and its antimicrobial effects against *Salmonella enterica* subsp. *enterica* serovar Typhimurium strain SL1344 in macrophage cells and in mice

**DOI:** 10.1186/s12906-018-2365-8

**Published:** 2018-12-05

**Authors:** Sung Phil Kim, Sang Jong Lee, Seok Hyun Nam, Mendel Friedman

**Affiliations:** 1 Research Institute of Basic Sciences, Ajou University, Suwon, 16499 Republic of Korea; 2 STR Biotech Ltd., Chuncheon, 24232 Republic of Korea; 3 Department of Biological Science, Ajou University, Suwon, 16499 Republic of Korea; 4 Western Regional Research Center, Agricultural Research Service, U.S. Department of Agriculture, Albany, CA 94710 USA

**Keywords:** Novel food formulation, Antimicrobial food, Salmonellosis, Autophagy, Phagocytosis

## Abstract

**Background:**

Human infection by pathogenic *Salmonella* bacteria can be acquired by consuming of undercooked meat products and eggs. Antimicrobial resistance against antibiotics used in medicine is also a major concern. To help overcome these harmful effects on microbial food safety and human health, we are developing novel antimicrobial food-compatible formulations, one of which is described in the present study.

**Methods:**

The composition of a bioprocessed (fermented) rice bran extract (BPRBE) from *Lentinus edodes* liquid mycelia culture was evaluated using gas chromatography and mass spectrometry, and the mechanism of its antibacterial effect against *Salmonella* Typhimurium, strain SL1344 was investigated in macrophage cells and in mice.

**Results:**

BPRBE stimulated uptake of the bacteria into RAW 264.7 murine macrophage cells. Activation of the cells was confirmed by increases in NO production resulting from the elevation of inducible nitric oxide synthase (iNOS) mRNA, and in protein expression. *Salmonella* infection down-regulated the expression of the following protein biomarkers of autophagy (a catabolic process for stress adaptation of cellular components): Beclin-1, Atg5, Atg12, Atg16, LC3-I and LC3-II. BPRBE promoted the upregulation of protein expressions that induced bacterial destruction in autolysosomes of RAW 264.7 cells. ELISA analysis of interferon IFN-β showed that inflammatory cytokine secretion and bactericidal activity had similar profiles, suggesting that BPRBE enhances cell-autonomous and systemic bactericidal activities via autophagic capture of *Salmonella*. The treatment also elicited increased excretion of bacteria in feces and their decreased translocation to internal organs (cecum, mesenteric lymph node, spleen, and liver).

**Conclusions:**

The antibiotic mechanism of BPRBE involves the phagocytosis of extracellular bacteria, autophagic capture of intracellular bacteria, and prevention of translocation of bacteria across the intestinal epithelial cells. The new bioprocessing combination of mushroom mycelia and rice brans forms a potentially novel food formulation with in vivo antimicrobial properties that could serve as a functional antimicrobial food and medical antibiotic.

## Background

*Salmonella* serotypes are reported to be the leading cause of foodborne disease outbreaks in the United States [1]. Rice plants produce bioactive rice brans and hulls, and mushrooms produce bioactive polysaccharides and other compounds. These food components have been reported to have numerous potential health benefits in cells, rodents, and humans, reviewed by Friedman [2–4]. The exposure of mushroom mycelia and rice bran to bioprocessing seems to result in the production of new bioactive compounds not present in the same combination that has not been bioprocessed. Our previously published studies have investigated the properties of rice hull smoke, rice brans, and bioprocessed (fermented) mushroom mycelia with added black rice bran, turmeric, and elm tree bark and will be briefly described here.

We previously reported that a liquid rice hull smoke extracts prepared by pyrolysis of rice hulls and liquefaction of the smoke [5] inactivated antibiotic-resistant *Salmonella* Typhimurium strains [6, 7] and protected mice against diabetes [8, 9], endotoxemia [10], and obesity [11]. We also reported that rice brans and their bioactive component γ-oryzanol protected mice and exhibited anti-allergic [12, 13] and anticarcinogenic properties in cells and in mice [14–18]. In other studies we showed that bioprocessed mushroom mycelia containing black rice bran [19], or turmeric [20] protected mice against salmonellosis; and a polysaccharide isolated from a liquid culture of mushroom mycelia containing black rice bran protected mice against endotoxemia [21]. In addition, mushroom mycelia bioprocessed with elm tree bark protected mice against allergic asthma [22].

The main objective of the present study was to elucidate the mechanisms that seem to govern the inactivation of foodborne *Salmonella* Typhimurium pathogenic bacteria by extracts of bioprocessed mushroom mycelia with added rice bran in murine macrophage cells and in mice and to relate the composition of the extracts, as determined using gas chromatography/mass spectrometry (GS/MS), to their inhibitory activity.

## Methods

### Materials

RPML-1640 medium (DMEM), phosphate-buffered saline (PBS), fetal bovine serum (FBS), and other cell culture reagents were purchased from Hyclone Laboratories (Logan, UT, USA). Potato dextrose agar medium (PDA), nutrient agar (NA) and MacConkey agar medium were the products from Difco Laboratory (Detroit, MI, USA). 3-(4,5-Dimethylthiazol-2-yl)-2,5-diphenyl tetrazolium bromide, MTT and other analytical grade reagents were obtained from Sigma-Aldrich (St. Louis, MO, USA).

### Preparation of bioprocessed rice bran extract (BPRBE)

The genetic identity of *Lentinus edodes* was confirmed by the Korean Center of Microorganisms (Seoul, Korea). Cultivation of the *Lentinus edodes* mushrooms and the bioprocessing of rice bran with added fungal mycelia were conducted according to our previously reported method [21, 22]. Briefly, the main liquid medium (3 L) containing rice bran (20 g/L) and dried soybean powder (2 g/L) was inoculated with the inoculum (10%) culture mycelia. The main liquid culture was then bioprocessed using 5 L fermenter at 28 °C and 150 rpm. After 7 days, the culture mass was ground in a colloid mill and the powder was then treated with 0.1 M lactic acid for 60 min, followed by treatment with an enzyme mixture for cell wall lysis. The acid- and enzyme-treated culture mass was then extracted with hot water at 90 °C and freeze-dried to a solid material. Non-bioprocessed rice bran extract (NPRBE), not subjected to the fungal mycelia fermentation but subjected to the acid and enzyme treatments, was used as a control formulation.

### Component analysis by GC-MS

The dried extracts from above were derivatized using previously published methods [22, 23]. Briefly, samples were reacted at 60 °C for 1 h with 100 μL of methoxyamine hydrochloride in pyridine (20 μL/mL). The sample was then silylated with 100 μL of *N*-methyl-*N-*trimethylsilyltrifluoroacetamide (MSTFA) (70 °C/1 h). GC/MS conditions were as follows: the gas chromatograph, model 6890GC (Agilent Technologies, Santa Clara, CA, USA) was equipped with a mass spectrometer detector 5975 and an apolar stationary-phase DB-1 column (100% dimethylpolysiloxane); (30 m × 0.25 mm; i.d. 0.25 μm film thickness). The starting oven temperature of the column at the time of injection, 70 °C, was maintained for 4 min and then ramped up at a rate of 10 °C/min until it reached a final temperature of 300 °C. The carrier gas (helium) had a flow rate of 1 mL/min. The injector and the detector were maintained at 250 °C. Injection volume was 1 μL, after being split 25 to 1. The mass spectrometer was set to electron ionization mode (70 eV). Identification of components was by both retention time, and comparison of the mass spectra to a commercial library [24].

### Bacterial strain and culture conditions

*Salmonella enterica* subsp. *enterica* serovar Typhimurium (*S.* Typhimurium) strain SL1344 was obtained from the National Collection of Type Cultures (Salisbury, Wiltshire, UK) and kept as frozen glycerol stock. The bacterial cell cultures were used as previously described [20].

### In vitro bactericidal assay

The bactericidal effect against *S*. Typhimurium SL1344 in liquid culture was examined as follows: aliquots of BPRBE and NPRBE were added to 3 mL *Salmonella* culture to form three concentrations of BPRBE (1, 10, and 100 μg/mL, respectively). The assay was performed using an inoculum size of *Salmonella* at 1 × 10^7^ colony-forming units (CFU). These were then allowed to grow for 12 h at 37 °C in nutrient broth. Cell growth profiles were determined spectrophotometrically by monitoring the turbidity of the culture at 600 nm at 2 h intervals for up to 8 h using a U*V*/VIS spectrophotometer (iMark™, Bio-Rad, Hercules, CA, USA).

### Mammalian cell culture

Murine RAW 264.7 macrophage cells were obtained from the American Type Culture Collection (Manassas, VA, USA). The cells were cultured in RPMI-1640 medium supplemented with 10% heat-inactivated FBS (*v*/v), 100 U/mL penicillin, and 100 μg/mL streptomycin. The cells were maintained at 37 °C with 5% CO_2_ in a humidified atmosphere.

### Cell viability assay

MTT staining as described by Mosmann [25], and previously used in this laboratory [22, 23], was used to evaluate the cytotoxicity of BPRBE and NPRBE. RAW 264.7 cells were seeded into 96-well tissue culture plate at a density of 1 × 10^4^ cells/well and cultured at 37 °C in humidified air with 5% CO_2_ for 16 h. The cells were then treated with 3 concentrations of BPRBE and NPRBE (1, 10, and 100 μg/mL) for 48 h and stained with added MTT. Absorbance was read in a microplate reader (iMark, Bio-Rad, Hercules, CA, USA) at 570 nm, with a reference wavelength of 655 nm. Cell viability was expressed as the percentage of live cells relative to those in the control group treated with PBS.

### Nitric oxide (NO) generation assay

Nitric oxide (NO) formation was measured by determining the concentration of its stable metabolite nitrite using a microplate assay as described by Murakami, et al. [26], a method previously used in this laboratory [23, 27]. RAW 264.7 cells (1 ×  10^5^ cells/well) in a 96-well tissue culture plate were treated simultaneously with lipopolysaccharide (LPS; 100 ng/mL) and either BPRBE or NPRBE at three concentrations (1, 10 and 100 g/mL each) for 48 h. After incubation, the culture medium was mixed with an equal volume of Griess reagent (1% sulfanilamide and 0.1% N-[1-naphthyl]ethylenediamine dihydrochloride in 5% phosphoric acid) at room temperature for 15 min. The absorbance was then measured at 570 nm using a microplate reader against a standard of sodium nitrite.

### Phagocytotic uptake assay

To measure internalized bacteria in macrophages, RAW 264.7 cells were infected with the *S.* Typhimurium as described by Lu, et al. [28]. To analyze the efficiency of bacterial uptake by macrophages, inoculum containing 1 × 10^4^ CFU was added to macrophage cells (1 × 10^4^ cells) pre-treated with three concentrations of BPRBE and NPRBE (1, 10, and 100 μg/mL) for 4 h, and then incubated for 60 min at 37 °C in humidified air with 5% CO_2_. Cells were washed once with RPMI-1640 medium after incubation and then treated to kill extracellular bacteria with the same medium containing 10% FBS and gentamycin (30 μg/mL) for 30 min. For viable cell counting, the infected macrophage cells were washed thrice and then lysed with sterile distilled water. Aliquots of lysates were then plated onto nutrient agar (NA) medium to measure CFUs. Phagocytosis efficiency of BPRBE and NPRBE was expressed as the fold increase of internalized bacteria relative to those without sample treatment.

### Measurement of interferon (IFN)-β

After RAW 264.7 cells (1 × 10^5^ cells) pre-treated with BPRBE and NPRBE (100 μg/mL) for 24 h were subsequently infected with *S.* Typhimurium (1 × 10^5^ CFU) for 5 h, the cell-free culture medium was recovered and stored at − 20 °C until use. IFN-β release into the culture medium was measured using an ELISA kit (PBL Assay Science, Piscataway, NJ, USA). The absorbance of the reaction mixture at 420 nm was measured in a microplate reader.

### Gastroenteritis animal model

Gastroenteritis was stimulated in test animals as described in a previous study [7]. Pathogen-free female Balb/c mice, aged 6 to 8 weeks, were purchased from Orient Bio Inc. (Seoul, Republic of Korea) and were hosted in a stainless-steel cage. Conditions were as follows: the light/dark cycle repeated every 12 h; the temperature was maintained at 20–22 °C; and the relative humidity was maintained at 50 ± 10%. The mice were given free access to a pelleted commercial chow diet (catalog no. 5 L79; Orient Bio Inc.). Tap water was available ad libitum. After a one-week acclimation, mice were divided into control and experimental groups (*n* = 10), avoiding any intergroup difference in body weight. Test groups were administered BPRBE or NPRBE (10 mg/kg) via oral dietary intake for 2 wk. Gastroenteritis was stimulated in mice as described by Barthel, et al. [29]. Briefly, 4-h fasting mice were administered streptomycin (20 mg in 75 μL water) by oral gavage. After 24 h, diet and water were again withdrawn for 4 h. Mice were then infected with *S*. Typhimurium strain SL1344 in 100 μL PBS (1 × 10^8^ CFUs) by oral gavage. After an additional 2 h of fasting, food and water was offered. The mice were monitored for 48 h and then sacrificed by asphyxia with CO_2_ to remove organs. The protocol for the mouse studies was approved by the Ethics Committee for Animal Care and Use, Ajou University, Suwon, Republic of Korea.

### Determination of bacterial load in organs and feces

Bacterial infection of the organs, and the presence in the feces, was determined as described in a previous study [7]. The cecum, mesenteric lymph node (MLN), spleen, and liver of the sacrificed animals were removed aseptically. The cecum was used after removing its content. The organs were weighed and then homogenized in 1 mL of sterile PBS with the aid of a tissue homogenizer maintained at 4 °C. Diluents of the homogenates were processed to count MacConkey agar medium supplemented with streptomycin (50 g/mL).

Fecal samples were collected at days 1–2 after gavage of *S.* Typhimurium in the mice from the *Salmonella* gastroenteritis model. The number of bacteria per gram of feces was determined as follows: Aliquots (100 μL) of fecal suspensions were serially diluted in PBS and then plated on the streptomycin-supplemented MacConkey agar medium. Cells were counted following incubation overnight at 37 °C.

### Reverse transcription polymerase chain reaction (RT-PCR)

Total cellular RNA was extracted using an Acid Phenol GTC-chloroform method as described by Chomczynski and Sacchi [30], and previously employed in this laboratory [22, 23, 27]. For RT-PCR, total RNA (1 μg) was incubated with AMV reverse transcriptase (6 U) and oligo (dT18) as primer (100 ng). DNA amplification from the iNOS gene was then primed in a reaction mixture containing dNTP mix (400 μM), *Taq* polymerase (2.5 U) and primer set (20 μM each): sense primer, 5’-ATGCCGAAGCAAACATCAC-3′; antisense primer, 5’-TAATGTCCAGGAAGTAGGTG-3′. Amplification was conducted in a thermocycler (PTC-200, MJ Research Inc., Reno, NV, USA) using the following program: one cycle for 5 min at 94 °C, 30 cycles for 30 s at 94 °C, 45 s at 58 °C, and 45 s at 72 °C, and finally one cycle for 5 min at 72 °C. The DNA was then fractionated with agarose gel electrophoresis and quantified using a gel documentation system (model LAS-100CH, Fuji Photo Film Co., Tokyo, Japan).

### Antibodies

Western blot analyses were performed using the following antibodies: rabbit anti-mouse iNOS polyclonal antibody from Santa Cruz (Dallas, TX, USA) and mouse anti-Actin monoclonal antibody from Millipore Corp. (Billerica, MA, USA), and all rabbit monoclonal antibodies raised against Beclin-1, Atg5, Atg12, Atg16L, LC3A/B, and phospho-IRF-3 from Cell Signaling Tech. (Danvers, MA, USA).

### Western blot analysis

The Western Blot technique was the same as described in previous studies [22, 23]. The BPRBE- and NPRBE-treated RAW 264.7 cells were lysed to extract total cellular proteins using RIPA buffer (50 mM Tris Cl, 150 mM NaCl, 15 NP-40, 0.5% sodium deoxycholate, 0.1% SDS and 1 mM EDTA, pH 7.4). Protein was quantified using a Bio-protein Kit (Bio-Rad) with bovine serum albumin (BSA) as a standard. Proteins from the cell extract (30 μg) were separated on 10% SDS-polyacrylamide gels and electrophoretically transferred onto the Millipore nitrocellulose membrane. After blocking with 5% skim milk, the membrane was incubated with primary antibodies to react for at least 3 h. The secondary antibody reaction with HRP-conjugated anti-IgG antibody was then performed under the same conditions. Blots were developed using the ELC detection kit (Pierce, Rockford, IL, USA). Quantification was achieved using a gel documentation system (model LAS-100CH, Fuji Photo Film Co., Tokyo, Japan).

### Statistical analysis

Analyses were run in triplicate, and expressed as the mean ± SD. Significant differences between means were determined by the ANOVA followed by Tukey’s test using the Statistical Analysis Software package SAS (Cary, NC, USA). *p* < 0.05 is regarded as significant.

## Results

### The composition of BPRBE and NPRBE

GC/MS analysis revealed that the two extracts contained 45 characterized compounds (Table 1). Some components remained unidentified, and others were tentatively identified. Among the identified compounds, 25 compounds were found solely in BPRBE and 13 structurally different compounds were only in NPRBE; seven compounds were present in both BPRBE and NPRBE. These results suggest that bioprocessing induces the formation of new compounds, probably catalyzed by enzymes present in the fungal mycelia. These compounds might be responsible for any observed antimicrobial activity of BPRBE.

**Table 1:** Compounds identified by GC/MS analysis of NPRBE and BPRBE

Peak	R.T. (min.)	Compounds	NPRBE	BPRBE
1	9.0	Propionic acid	–	1.76
2	10.0	L-Valine	–	0.06
3	10.4	1-(3-Methylbutyl)-2,3,4,6-tetramethylbenzene	0.29	–
4	12.2	L-Leucine	–	0.14
5	12.4	Butanoic acid	–	0.16
6	15.7	Cadaverine	–	0.06
7	16.0	Glycerol	6.29	2.57
8	16.8	Glycine	–	0.07
9	17.2	Butanedioic acid	–	0.43
10	17.8	Pyrimidine	–	0.05
11	18.3	Fumaric acid	–	0.05
12	19.6	Hydroquinone	–	0.02
13	20.3	L-Aspartic acid	0.11	–
14	22.1	Hexadecane	–	0.08
15	22.6	Erythritol	–	0.26
16	22.6	D-(+)-Arabitol	0.04	–
17	22.8	Proline	0.07	0.07
18	22.9	L-Aspartic acid	0.02	–
19	23.4	Methyl 2-amino-4-(2-aminophenyl)-4-oxobutanoate	–	0.11
20	24.2	3-Pyridinecarboxylic acid	–	0.10
21	25.6	Xylonic acid	–	0.06
22	26.4	D-Glucose	0.17	0.09
23	26.7	D-Xylose	–	0.59
24	27.7	Xylitol	0.07	–
25	28.7	Phosphoric acid	0.35	–
26	29.5	Azelaic acid	0.08	–
27	30.0	Citric acid	0.21	–
28	30.6	D-Glucuronic acid	0.06	–
29	31.3	Ribitol	–	0.28
30	31.3	D-(+)-Gluconic acid	0.05	–
31	31.56	D-Glucose	0.18	–
32	31.63	D-Galactose	–	55.51
33	32.0	D-Mannose	–	10.08
34	32.4	D-Mannitol	0.05	–
35	32.8	Gluconic acid, 1,4-lactone	–	0.18
36	33.7	D-Gluconic acid	0.25	–
37	34.3	Glucopyranose		0.76
38	34.7	Hexadecanoic acid	0.07	0.09
39	35.4	Myo-inositol	0.52	0.44
40	38.4	Stearic acid	0.03	0.05
41	39.4	2-O-Glycerol-α-d-galactopyran		0.14
42	41.8	Nonanol		0.02
43	44.9	Sucrose	86.42	0.40
44	46.5	[4-Bromo-2-2-hysrazono-phenyl-methyl)-phenyl]-carbamic acid, ethyl ester-		13.32
45	46.7	Maltose		0.13

*BPRBE* bioprocessed rice bran extract, *NPRBE* non-processed rice bran extract

### BPRBE is not bactericidal against *Salmonella* in PBS buffer

**Fig. 1:**
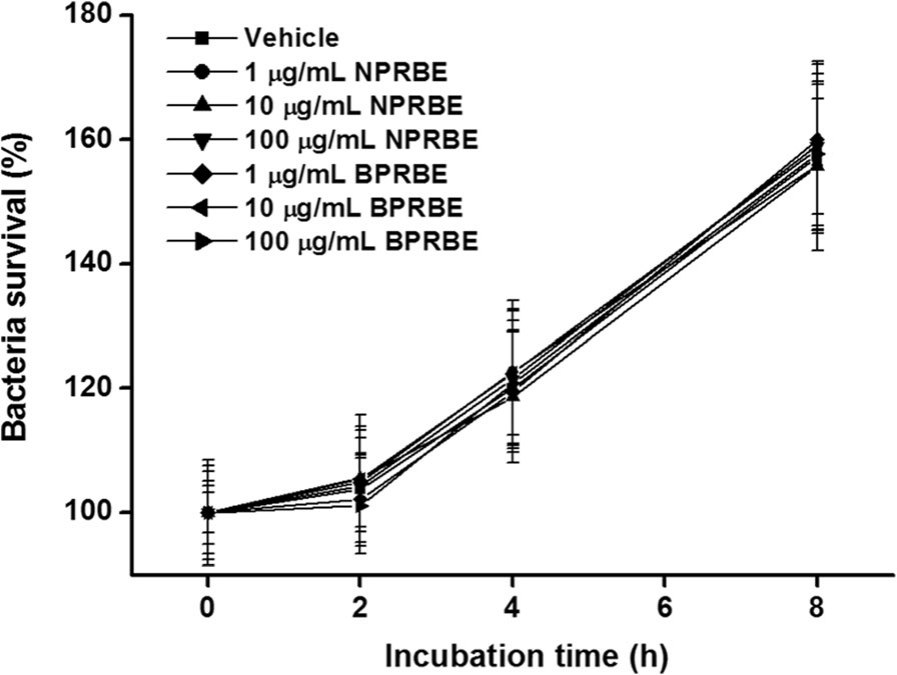
Effects of BPRBE and NPRBE on growth of *S*. Typhimurium. Serially diluted BPRBE and NPRBE (1, 10, and 100 μg/mL) were incubated with *S*. Typhimurium (2 × 10^4^ CFU) for 2, 4, 6, and 8 h. Plotted values are mean ± SD of triplicate experiments

To find out if the extract has bactericidal activity against *S*. Typhimurium, the bacteria were incubated with serially diluted BPRBE in PBS (1, 10, and 100 μg/mL) at 37 °C for 0, 2, 4, 6, and 8 h. Fig. 1 shows that that there was no significant bactericidal activity up to 8-h of incubation. The data demonstrate that BPRBE did not kill the bacteria directly. We previously observed a similar lack of bactericidal activity in vitro by a polysaccharide isolated from a black rice bran extract bioprocessed with mushroom mycelia [19].

Because the chemical nature of the identified compounds shown in Table 1 varied widely, it is difficult to determine which compound or combination of compounds might be responsible for the bioactivity of BPRBE. This aspect merits further study.

### Effects of BPRBE on phagocyte activation and cell survival

To examine whether BPRBE can enhance cell-mediated immune function, RAW 264.7 murine macrophage cells were cultivated in the presence of the extracts, and the resultant NO production was then assessed as an indicator of macrophage activation. Table 2 shows that BPRBE (1, 10, and 100 μg/mL) markedly induced NO production in a dose- dependent manner. The NO formation levels of BPRBE at 10 μg/mL or higher doses were significantly higher than that obtained with lipopolysaccharide (LPS) (100 ng/mL). By contrast, NPRBE used as internal control barely induced NO production relative to the vehicle.

**Table 2:** Effects of NPRBE and BPRBE on macrophage activation and survival

Sample	Nitrite (μM)	Cell viability (%)
Vehicle	2.02 ± 0.20^f^	100.0 ± 1.8^a^
LPS	31.13 ± 0.34^c^	–
NPRBE (μg/mL)		
1	1.80 ± 0.20^f^	102.3 ± 2.8^a^
10	1.75 ± 0.60^f^	102.9 ± 2.2^a^
100	6.91 ± 0.67^e^	102.7 ± 2.9^a^
BPRBE (μg/mL)		
1	23.62 ± 0.56^d^	103.6 ± 3.4^a^
10	34.4 ± 1.2^b^	102.5 ± 1.7^a^
100	39.3 ± 1.2^a^	100.3 ± 2.5^a^

Data are expressed as the mean ± SD (*n* = 3). Values in each column with the same letter are not significantly different between groups at *p* < 0.05. BPRBE, bioprocessed rice bran extract; NPRBE, non-processed rice bran extract

**Fig. 2:**
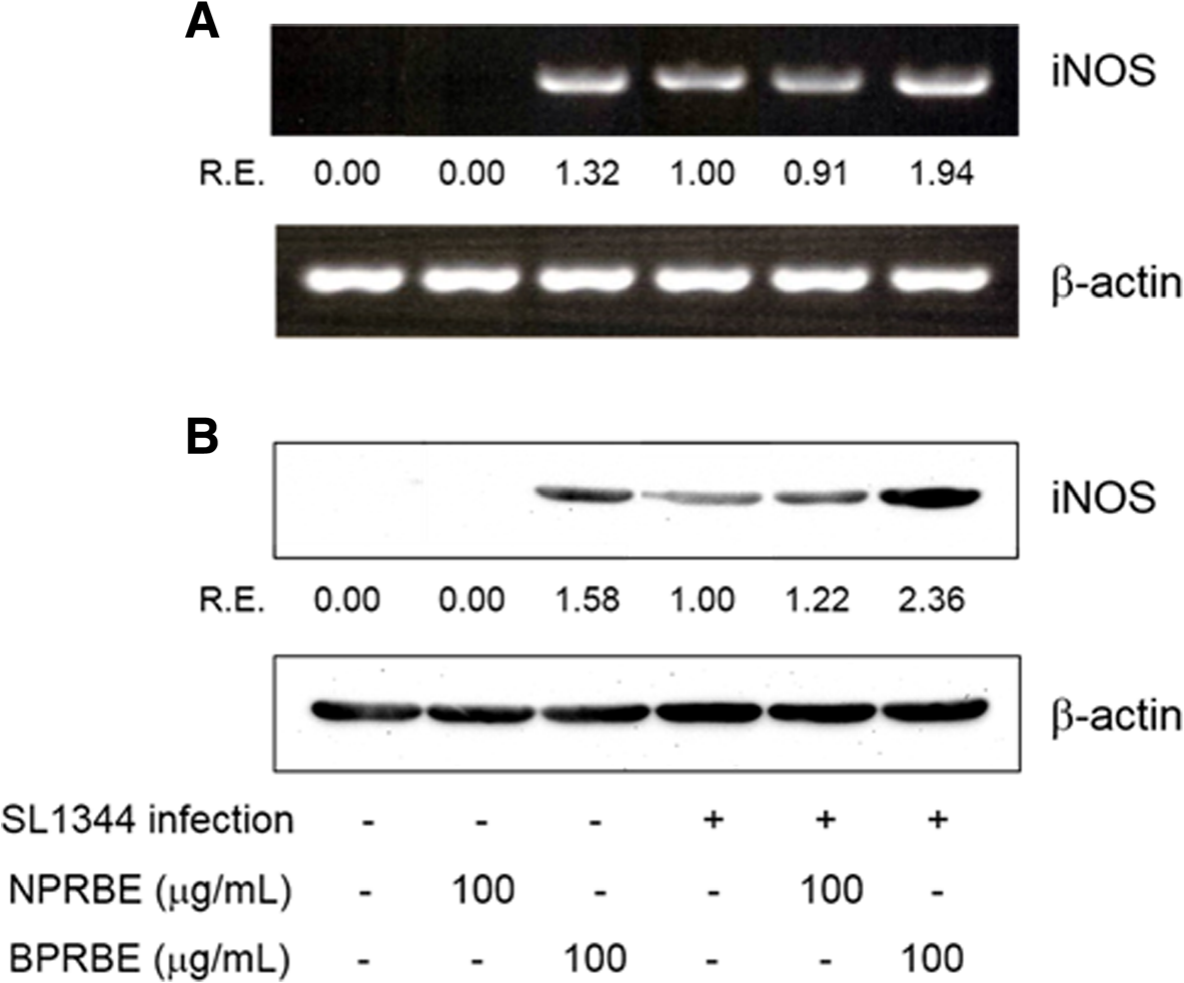
Effects of BPRBE on iNOS gene expression in *S.* Typhimurium-infected macrophages. **a** iNOS mRNA expression profiles assessed by RT-PCR. RAW 264.7 cells were cultivated with or without BPRBE for 24 h. The cells were then infected with *S.* Typhimurium SL1344 for 8 h. After infection, total RNA was purified and an iNOS mRNA expression profile was determined using RT-PCR analysis. **b** Assessment of iNOS protein expression profiles by Western blot analysis. RAW 264.7 cells treated with BPRBE for 24 h and then infected with *S.* Typhimurium were lysed and the iNOS protein in the cell lysate was then identified by Western blotting using rabbit anti-mouse iNOS polyclonal antibody. The relative proportions of iNOS mRNA and polypeptide are expressed as the R. E. (relative expression) values calculated from iNOS/β-actin gene expressions. Figures represent results from at least three individual experiments

RT-PCR was used to confirm BPRBE-induced NO production at the gene expression level. Fig. 2a shows that the mRNA expression level from the iNOS gene of *Salmonella*-infected cells pre-treated with BPRBE was ~ 1.9-fold higher than that in the bacteria infected cells without treatment by the extract. Western blot analysis also shows that the iNOS protein expression profile was similar to that of mRNA expression (Fig. 2b). These data indicate that bioprocessing induces the activation of immune function in the macrophages.

Next, the cytotoxicity of the BPRBE treatments on RAW 264.7 cells was evaluated using the MTT assay. The results show that at three doses, survival rates of the BPRBE- and NPRBE-treated cells were not statistically different from those of the vehicle-treated control, indicating that BPRBE was not toxic to cells.

### Effects of BPRBE on the stimulation of phagocytosis

**Fig. 3:**
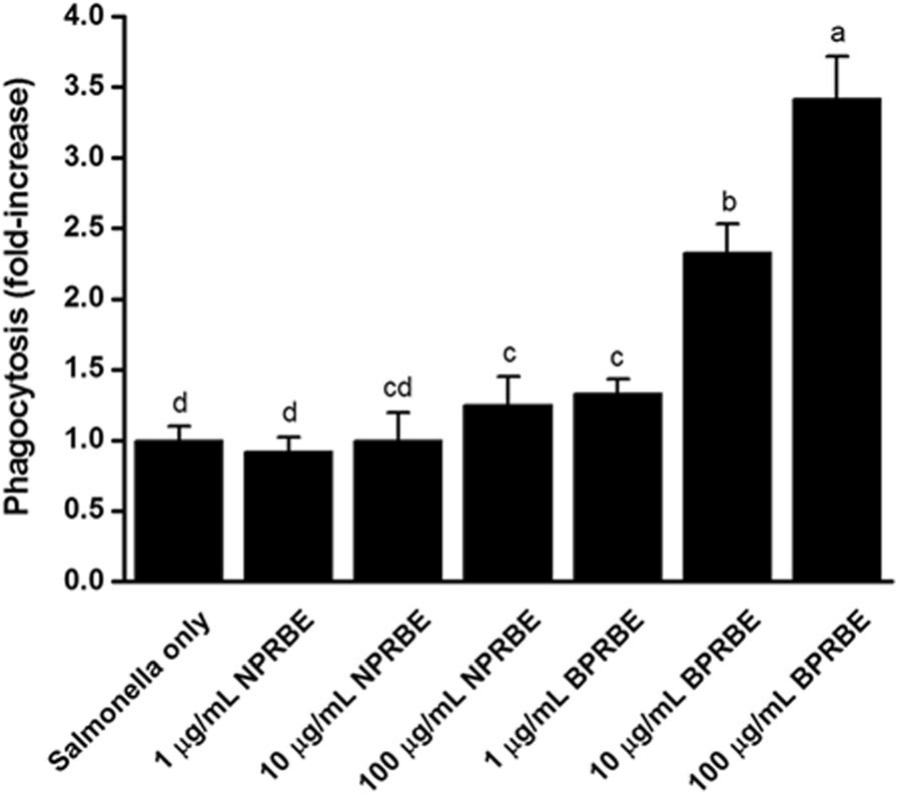
Dose-dependent change in the phagocytotic effects of BPRBE on *S.* Typhimurium-infected macrophages. RAW 264.7 cells (1 × 10^4^ cells) were incubated with three concentrations of BPRBE and NPRBE (1, 10 and 100 μg/mL) for 4 h and were then infected with *S.* Typhimurium SL1344 (1 × 10^4^ CFU). After incubation for 60 min, bacteria-infected macrophages were cultivated in the presence of gentamycin (30 μg/mL) for 30 min. Bacterial internalization efficiency was determined by measuring the protection of internalized bacteria against bactericidal action of the antibiotic gentamycin. Data are expressed as the mean ± SD of triplicate experiments. Bars sharing a common letter are not significantly different between groups at *p* < 0.05

To examine if BPRBE indirectly exerts bactericidal activity through phagocytosis, RAW 264.7 cells pre-treated with BPRBE at three doses were infected with *S.* Typhimurium SL1344 bacteria, followed by cell lysis and enumeration of their content. Fig. 3 shows that internalization of *Salmonella* into the macrophage cells was increased by the BPRBE treatment in a dose-dependent manner. The *Salmonella* internalization rates in the macrophages treated with 1, 10 and 100 μg/mL BPRBE for 4 h were about ~ 1.3-, 2.3-, and 3.4-fold greater than the rate with the vehicle-treated control. By contrast, NPRBE failed to increase the bacterial internalization at a significant level, even at the highest concentration of 100 μg/mL. These results indicate that the bioprocessing of rice bran with a mushroom mycelia culture induces bactericidal activity in the macrophage cells by enhancing their phagocytic potential in a dose-dependent manner. These results are similar to the observations by Kim, et al. [31] on the antibacterial activity of the natural herb *Houttuynia cordata* on *Salmonella* within RAW 264.7 macrophage cells.

### Effect of BPRBE on autophagy

**Fig. 4:**
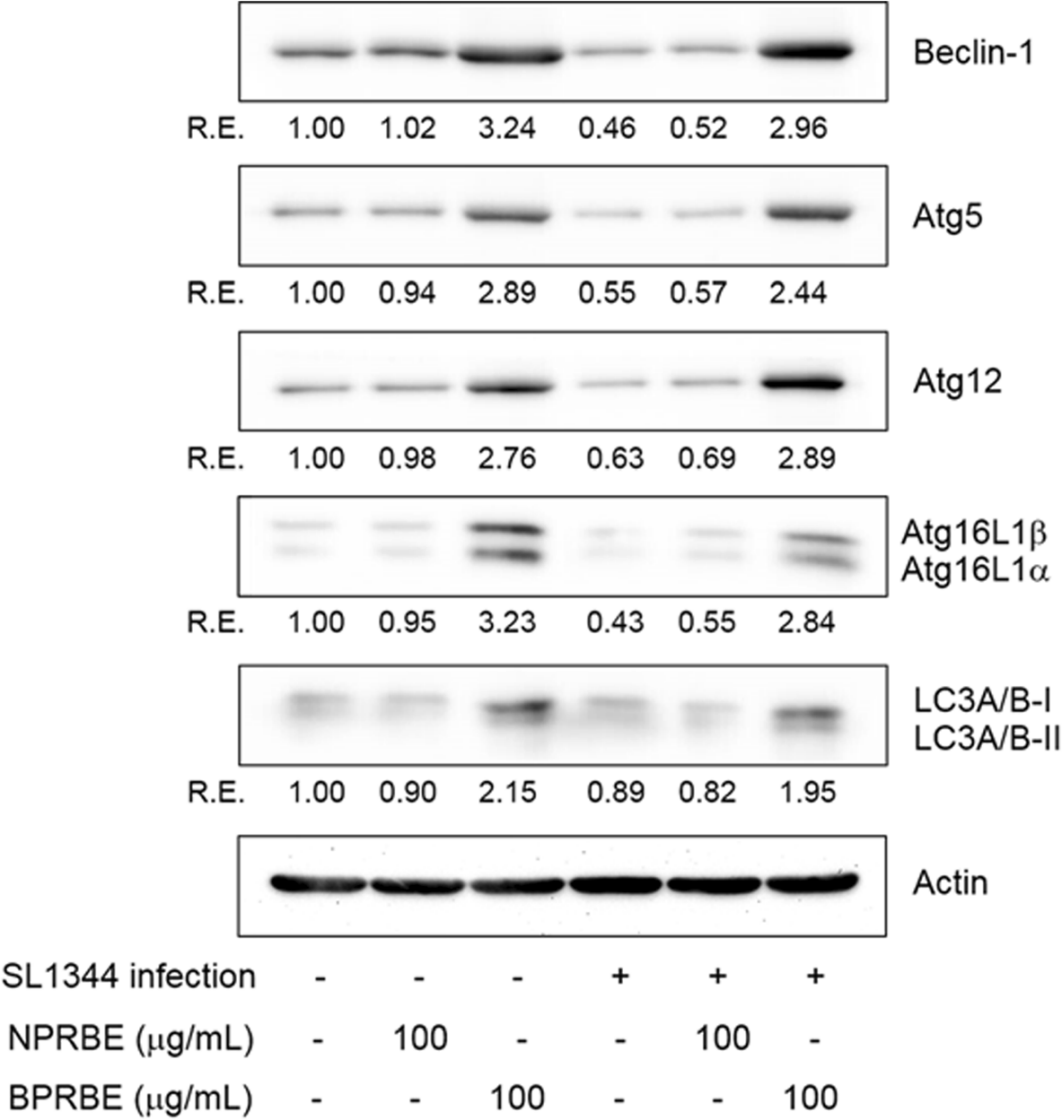
Effects of BPRBE on induction of Beclin-1, Atg5, Atg12, Atg16L and LC3 in *S.* Typhimurium-infected macrophages. RAW 264.7 cells were lysed after treatment with BPRBE and NPRBE (100 μg/mL) for 24 h and infected with *S.* Typhimurium for 5 h. The cell lysate was subjected to electrophoresis on 12% SDS-PAGE. Western blotting was carried out with the primary antibodies with β-Actin as an internal control. The relative proportions of each protein are expressed as the R. E. (relative expression) values calculated from each target protein/β-Actin expression. Figures represent results from at least three individual experiments

Many intracellular pathogens including *Salmonella* have evolved strategies that suppress autophagy, or evade autophagic recognition, leading to intracellular bacterial replication in a *Salmonella*-containing vehicle (SCV). The following experiments were carried out to determine if BPRBE can enhance autophagy in RAW 264.7 cells. The expression of Beclin-1, Atg5, Atg12, Atg16L proteins, microtubule-associated (LC3-I), and membrane-associated (LC3-II) light chain 3, which are needed for autophagic vacuole formation, phagophore membrane elongation, and autophagosome formation, respectively, were assessed in uninfected and *Salmonella*-infected RAW 264.7 cells in the absence or presence of BPRBE using Western blot analysis. The results show that *Salmonella* infection down-regulated expression of the autophagy-related proteins including Beclin-1 compared with the uninfected controls (Fig. 4). However, the BPRBE treatment up-regulated the expression of these autophagy-related proteins regardless of bacterial infection. These results indicate that the BPRBE treatment might block the escape of *Salmonella* from autophagy by the up-regulation of the expression of proteins involved in the assembly of the autophagic machinery, leading to cell-autonomous bactericidal action, i.e. direct bacterial destruction in the autolysosomes [32].

### The effect of BPRBE on IFN-β production

**Fig. 5:**
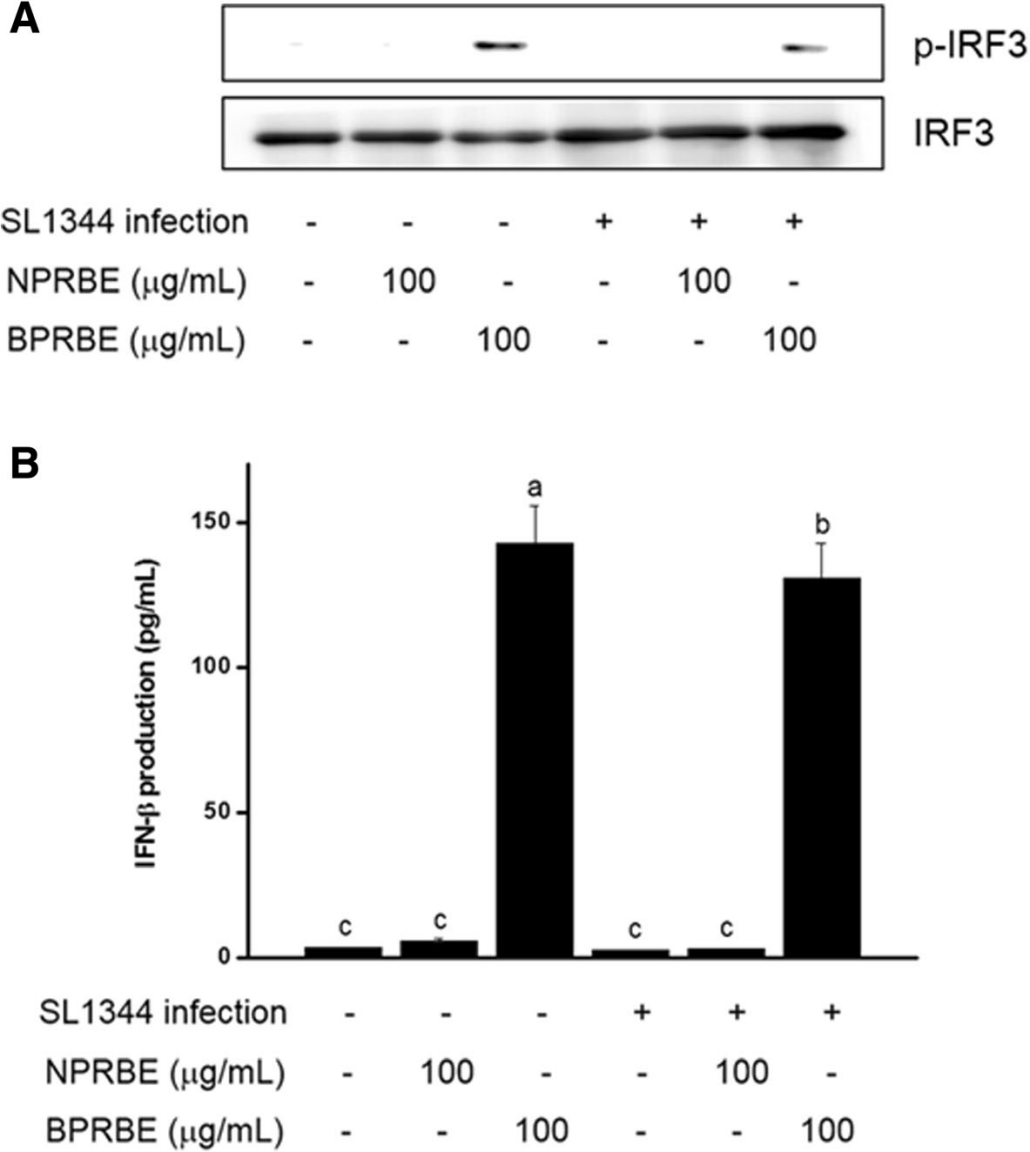
Effects of BPRBE on induction of IFN-β production through IRF3 activation in *S.* Typhimurium-infected macrophages. **a** Phospho-IRF3 protein (p-IRF3) expression profiles assessed by Western blot analysis. RAW 264.7 cells (1 × 10^5^) were treated with BPRBE and NPRBE for 24 h and infected with *S.* Typhimurium and lysed. The phospho-form of IRF3 in the cell lysates was identified using Western blotting. **b** IFN-β release from *S.* Typhimurium-infected macrophages pre-treated with BPRBE and NPRBE was determined using an ELISA kit. Data are expressed as the mean ± SD of triplicate experiments. Bars sharing a common letter are not significantly different at *p* < 0.05

The autophagic capture of *Salmonella* is known to enhance not only cell-autonomous bactericidal action, but also systemic mechanisms of bacterial killing initiated by type I interferon production. It has been shown that the release of bacterial degradation products leads to activation of endosomal Toll-like receptors (TLRs), which produce IFN-β through the activation of the interferon regulatory transcription factor IRF-3 [33]. Therefore, to determine whether the BPRBE treatment can enhance IFN-β production via activation of the IRF-3-mediated signaling pathway in *Salmonella*-infected RAW 264.7 cells, IFN-β release and IRF-3 activation were assessed by ELSA and Western blot analysis. Fig. 5 shows that the BPRBE treatment markedly induced phosphorylation of IRF-3 leading to the concomitant production of IFN-β in the *Salmonella*-infected cells. In contrast, the NPRBE treatment failed to elicit both IRF-3 activation and IFN-β production. These results show that BPRBE can restore the systemic bactericidal mechanism to normal status in terms of IFN-β production from a suppressed level by blockading the escape of the infected bacteria from autophagic recognition.

### The effects of BPRBE on bacterial translocation to internal organs

An in vivo assay using gastroenteritis model mice was used to examine if dietary administration of BPRBE suppresses the translocation of *S*. Typhimurium to internal organs through the intestinal epithelial barrier. Table 3 shows that the number of bacteria excreted in the feces increased ~ 1.7- to 2-fold with BPRBE relative to the control on day 1 or 2 after infection with *S.* Typhimurium. Additional experiments on the translocation of *S*. Typhimurium to internal organs of infected mice show that BPRBE administration reduced bacterial load in the cecum, mesenteric lymph node, spleen, and liver 2 days after infection by ~ 56, 75, 94, and 88%, respectively. NPRBE showed only insignificant effects on bacterial translocation to the organs compared with the control. These results suggest that BPRBE can suppress the translocation of *S*. Typhimurium to internal organs through the intestinal epithelial cell barrier, and, in parallel, enhance autophagy-mediated cell-autonomous and systemic bactericidal action, thus limiting orally infected *S*. Typhimurium dissemination from the intestine to several organs.

**Table 3:** Effect of NPRBE and BPRBE on bacterial load in feces and organs from *Salmonella*-infected mice

	Treatment		
	*Salmonella* infection only	NPRBE (10 mg/kg)	BPRBE (10 mg/kg)
*Salmonella* in feces (× 10^5^ CFU/g)			
1 day	338 ± 18^b^	347 ± 21^b^	662 ± 48^a^
2 day	420 ± 31^b^	448 ± 35^b^	729 ± 53^a^
*Salmonella* in organs			
Cecum (× 10^5^ CFU/g)	35.3 ± 2.7^a^	30.6 ± 1.5^a^	15.4 ± 1.3^b^
Mesenteric lymph node (× 10^3^ CFU/organ)	34.7 ± 1.9^a^	31.4 ± 2.7^a^	8.8 ± 0.6^b^
Spleen (× 10^2^ CFU/organ)	172.1 ± 8.8^a^	165.3 ± 11.2^a^	10.5 ± 0.9^b^
Liver (× 10^2^ CFU/organ)	80.6 ± 7.6^a^	75.4 ± 2.9^a^	9.6 ± 0.7^b^

Data are expressed as the mean ± SD (*n* = 3). Values in each row with the same letter are not significantly different between groups at *p* < 0.05. BPRBE, bioprocessed rice bran extract; NPRBE, non-processed rice bran extract

## Discussion

The results of the present study show that the new food formulation produced by bioprocessing (fermenting) a mushroom mycelia culture supplemented with rice bran contains bioactive compounds not present in the same combination of mycelia and rice bran that was not bioprocessed. It is likely that the presence of certain enzymes in the culture catalyze the formation of the new compounds. Although extracts of the bioprocessed product did not inhibit the growth of *Salmonella* in an in vitro bactericidal assay in laboratory media, they did inhibit the growth of the pathogen in infected macrophage cells and in different mouse organs (cecum, mesenteric lymph node, spleen, and liver). Because previous studies showed that lipopolysaccharides (LPS) induce autophagy in hepatocytes in vitro and in rodents, it is possible that polysaccharides produced during the described fermentation process might be the active autophagic compounds [34–37]. However, we do not have specific evidence for this suggestion.

These results complement and extend our findings with similarly bioprocessed combinations of mushroom mycelia with turmeric [20] and elm tree bark [22]. The practical value of this approach is reinforced by the related observation by Han, et al. [38] that a bioprocessed polysaccharide from *Lentinus edodes* mycelia liquid culture with added turmeric protected chicks from a lethal challenge of *Salmonella* Gallinarum, suggesting that the formulation has the potential to serve as an antimicrobial livestock feed and possibly also as an antibiotic functional food in human diets.

The effect of the bioprocessed extract on the phagocytic response to bacterial infection has been investigated here, and the importance of autophagy in response to pathogens has been well documented, as indicated by the following selected observations. Yu, et al. [39] describe autophagy (self-eating in Greek) as a process whereby a double-membrane structure (autophagosome) engulfs unnecessary cytosolic proteins, organelles and invading pathogens and delivers them to the lysosome for enzymatic degradation. These authors reported that the depletion of autophagy components significantly reduced the replication of cytosolic *Salmonella* in Hela human epithelial cells. A study by Liang, et al. [40] shows that a Beclin-1 autophagy protein complex induces innate immune responses essential for eliminating pathogens. Kim, et al. [41] discusses how host cell autophagy orchestrates successful antimicrobial response in mycobacteria to antibiotics [41] and Wang, et al. [42] discuss how autophagy has been demonstrated to be an important defense mechanism to clear intracellular pathogenic organisms and as a process that regulates immune responses.

Bernal-Bayard and Ramos-Morales [43] review relevant studies on the molecular mechanisms used by *Salmonella* to evade the immune system by manipulating inflammatory pathways, Barakat and Friedman [44] describe the complex mechanism of autophagy in prostate cancer cells, and Nakahira, et al. [45] discuss the role of autophagy in the pathogenesis of lung diseases. These observations suggest that further investigation might focus on whether the mushroom mycelia-rice bran formulation could also inhibit the growth of cancer cells via autophagy.

## Conclusions

Here we have described the evaluation of a potential-health-promoting functional food, a mushroom mycelia-rice bran formulation. The orally administered formulation inhibited the growth of *Salmonella* Typhimurium in infected mouse organs. We elucidated the mechanism by which the formulation seems to govern the destruction of the foodborne pathogenic microorganism *Salmonella* Typhimurium in mice. In addition, detailed experiments described here using macrophage cells have helped to define the biomarkers associated with bactericidal activity.

Our efforts to investigate the possible mechanism of the antibiotic effects shows that phagocytosis against *Salmonella* Typhimurium in the macrophage cells and bactericidal activity in mice is associated with an enhanced autophagic activity of the macrophages, leading to macrophage-mediated bactericidal action as well as to systemic bactericidal action through type 1 interferon production. This mechanism seems to govern the inactivation of bacteria and prostate cancer cells mentioned earlier. Whether autophagy also governs the inactivation of disease-causing protozoa by natural compounds [46, 47] merits study.

Because foodborne and medical bacteria often resist the action of conventional antibiotics [48], it would be of interest to determine if the antimicrobial formulations produced via bioprocessing also inhibit the growth of multidrug-resistant *Salmonella* and other foodborne pathogens in infected rodents, as we observed with a rice hull smoke extract [7]. It would also be useful to determine if BPRBE could kill both pathogenic bacteria and cancer cells by the autophagy process. Clinical trials are needed to confirm the beneficial results in infected humans. This study is expected to stimulate future studies to confirm the antimicrobial health benefits of the new food product in animal feeds, human foods, and in humans.

Finally, it is of theoretical and practical interest that unlike BPRBE, other natural antimicrobials including plant essential oil compounds, winery byproducts, shellfish-derived chitosans, tea compounds, and fruit and vegetable peel powders induce destruction of pathogens both in vitro and in vivo by mechanisms involving the disruption of cell membranes and chemical modification of essential proteins and DNA [49–56]. In future investigations, BPRBE could be combined with other natural antimicrobials to determine if they could neutralize pathogens in animal feeds, human foods, and in humans.

## Abbreviations

ANOVA: Analysis of Variance
BPRBE: Bioprocessed Rice Bran Extract
BSA: Bovine Serum Albumin
CFU: Colony-Forming Units
ELISA: Enzyme-Linked Immunosorbent Assay
FBS: Fetal Bovine Serum
GC/MS: Gas Chromatography/Mass Spectrometry
HRP: Horseradish Peroxidase
IFN-β: Interferon Beta
iNOS: Inducible Nitric Oxide Synthase
IRF3: Interferon Regulatory Factor 3
mRNA: Messenger RNA
MSTFA: *N*-Methyl-*N*-Trimethylsilyltrifluoroacetamide
MTT: 3-(4,5-Dimethylthiazol-2-yl)-2,5-Diphenyl Tetrazolium Bromide
NA: Nutrient Agar
NO: Nitric Oxide
NPRBE: Non-Bioprocessed Rice Bran Extract
PBS: Phosphate-Buffered Saline
PDA: Potato Dextrose Agar Medium
RT: retention times
TLR: Toll-Like Receptor
